# The Effect of Distance Education Conducted during the COVID-19 Pandemic Period on the Psychomotor Skill Development of a Dental School Students

**DOI:** 10.1155/2022/6194200

**Published:** 2022-06-09

**Authors:** Erdal Eroğlu, Giray Kolcu, Mukadder İnci Başer Kolcu

**Affiliations:** ^1^Department of Prosthodontics, Süleyman Demirel University, Faculty of Dentistry, Isparta, Turkey; ^2^Department of Medical Education and Informatics, Süleyman Demirel University, Faculty of Medicine, Isparta, Turkey; ^3^Süleyman Demirel University Vice Director of Institute of Health Sciences, Isparta, Turkey

## Abstract

**Purpose:**

This study compared the development of face-to-face (F2F) psychomotor skills vs. a modified online dental anatomy course during the COVID-19 pandemic.

**Methods:**

The 9-HPT is regularly applied for the students' psychomotor skill assessment in the Faculty of Dentistry of Süleyman Demirel University. In the 2020-21 academic year, 92.2% of students who took the dental anatomy course in distance education voluntarily participated in this study (observation group). These data were compared with 34.6% of students who applied for 9-HPT in 2019-2020 in F2F education (control group). The Mann–Whitney *U* test and *t*-test were used for comparison. The significance level was taken as *p* < 0.05.

**Results:**

Based on the dominant hand 9-HPT score, a positive change occurred in 81.8% of the F2F and 61.6% of the online education students. Based on both dominant and nondominant hand 9-HPT scores, a statistically significant difference between the change in scores according to the fall/spring term measurements was observed in both F2F and distance education (*p* < 0.001). However, the F2F-educated group students showed better psychomotor skill development than those exposed to the online-educated group in 9-HPT. The results showed statistical significance for both dominant and nondominant hands (*p* < 0.001).

**Conclusion:**

The F2F education is the gold standard in psychomotor skill training; however, in cases where this approach cannot be realized, practical training protocols modified for online training may contribute to the motor development of students.

## 1. Introduction

Dentistry is a field of health that requires excellent motor skills, advanced hand-eye coordination, and spatial perception. In addition, perceptual learning is needed for indirect visualization through an intraoral mirror. The acquisition of these traits has been one of the main goals of dentistry programs worldwide as a scholarly output for students [1, 2]. These outputs were determined as part of the primary program output by the Council of Higher Education in Turkey (CoHE), and their importance in dentistry programs was stated [3].

Practical courses have been intensively presented from the beginning of the first semester in this country to acquire these skills individually. As in the rest of the world, the dental anatomy course occurs in the first academic year as a part of the core education program [4, 5]. The primary objectives of this preclinical course in the dental curriculum are to improve students' cognitive and psychomotor skills related to the morphology and spatial and functional relationships of human dentition and promote these skills to recreate a proper tooth form in restorative clinical procedures competently [6–8].

Terminologically, psychomotor skills define motor skills, hand skills, and 3D perception. These skills affect individuals' activities related to daily life and have a very significant impact on their professional competence [9, 10]. They can be measured using various hand skill tests to evaluate individuals' motor capacity or disability. These tests are often used in physical therapy and rehabilitation centers to evaluate professional development or determine the preoccupational condition. The most common of these evaluation tests include block carving, thermometer testing, O'Connor Tweezer Dexterity Test, and Purdue pegboard test. However, there is no consensus on the best one from a psychometric perspective [2]. The Nine Hole Peg Test (9-HPT) is a simple, fast, dexterity test with proven validity and reliability. It is susceptible to changes in upper extremity performance [11, 12]. In many dental curricula, initial psychomotor skill development assessment may not be possible until the students move on to preclinical courses. The evaluation of psychomotor competencies in the early period is essential to increase the clinical success of students [13–15].

Psychomotor skill tests can be used as an admission criterion in the dentistry program in some countries [16–19]. These tests may also help evaluate the applied courses' effect or compare the courses' success with counterparts in other dentistry programs [20]. Since 2018, 9-HPT has been applied to dentistry students (participation is voluntary) with the permission of the SDU ethics committee, and the results were also monitored.

Due to the COVID-19 pandemic, higher education institutions in Turkey have carried out distance education for a significant part of the 2020-2021 academic year [21]. As a part of this distance education protocol, the educators of the dental anatomy course at Süleyman Demirel University tried to conduct the applied dental anatomy courses online.

The present study compared the effect of the face-to-face course and a modified online dental anatomy course on psychomotor skill development via 9 HPT during the COVID-19 pandemic.

## 2. Materials and Methods

The study was designed as a pretest/posttest study with a control group in a quantitative research design and was carried out at Süleyman Demirel University Faculty of Dentistry in March 2021. Approval for the study was obtained from the Ethics Committee of Süleyman Demirel University and the Faculty of Dentistry (ethics committee approval date: 01.06.2021 and 51/9).

Of the 141 students involved in the F2F dental anatomy course in the 2019-2020 academic year, 121 (85.8%) applied for this test voluntarily. These data were included in the study as the control group. In the 2020-21 academic year, 47 (34.6%) of 136 students taking this modified online course participated in this test. The data of these students were also included in the study as an observation group.

**Figure 1:**
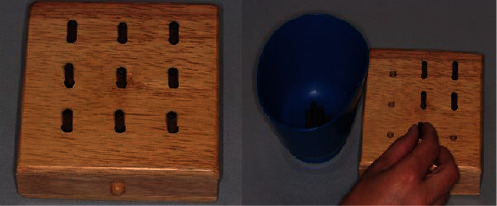
The 9-HPT kit and its application.

The 9-Hole Peg Test (9-HPT) is a brief, simple, standardized, timed measure test to evaluate upper extremity function and skills. 9-HPT material comprises a shallow dish next to the 9-hole pegboard with nine wooden/plastic pegs (Baseline Evaluation Instruments, USA, Lot: 055965) (Figure 1). The pegboard is placed before the subject at the midline, with the container holding the pegs oriented towards the participant's hand being tested. The subjects are seated on a height-appropriate chair to ensure that the tabletop is at midchest level. The test task requires the subject to take the pegs from a container one by one and place them into the holes on the board as quickly as possible. And then, they ought to remove the pegs from the holes and replace them in the container one by one (Figure 1). The time taken to complete the test activity in seconds is recorded as the score. The test begins with the dominant hand. Two consecutive trials with the dominant hand are followed by two consecutive trials with the nondominant hand. The means of the scores are recorded as dominant and nondominant hand scores. To avoid bias, the 9-HPT tests conducted in the 2019-2020 and 2020-2021 academic years were performed by the same observer.

In F2F education, volunteer students have performed this 9-HPT since the 2018-2019 academic year. The test was administered at the beginning of the fall and spring semesters to observe the psychomotor skill development of students. The psychomotor skill test in 2019-2020 (at the beginning of the fall and spring terms) was also carried out voluntarily under the same conditions. The scores of the 9-HPT in the 2019-2020 academic year were used as the control group to compare the psychomotor skills gained from the dental anatomy courses.

In the distance education group (observation group), due to the COVID-19 pandemic in the 2020-2021 academic year, the dental anatomy course started in October with distance (online) education. However, in the spring term of the 2020-2021 academic year (distance education during the COVID-19 pandemic), the Council of Süleyman Demirel University and the Administration of Faculty of Dentistry decided to return to the F2F training in April 2021 as the restrictions due to COVID-19 were relaxed. Due to this announcement, students were given a chance to participate in the F2F education. Consequently, after distance education, 51 of 136 students participated in the F2F dental anatomy course training. All the first-year students (*n* = 51) involved in the applied F2F course were interviewed to participate in the study. The students were informed about the study and were guaranteed that they would not be advantaged/disadvantaged by accepting/refusing to participate. 47 (*n* = 47) of them accepted to participate, and each participant signed a written consent form according to the World Medical Association's Helsinki Declaration. 9-HPT was applied for both dominant and nondominant hands of the participants (*n* = 47). The scores were recorded.

In the study's statistical analysis, descriptive analyses of the measurements were made, and the Mann–Whitney *U* test and *t*-test were used for comparison. The data were analyzed with IBM SPSS V23, and the significance level was taken as *p* < 0.05.

## 3. Results

The 9-HPT scores of first-year students in the Faculty of Dentistry of Süleyman Demirel University in the academic years of 2019-2020 and 2020-2021 (*n* = 121 and *n* = 47, respectively) were used as data in this study (Table 1).

Based on the dominant hand 9-HPT score, F2F education caused a positive change in 99 (81.8%) and an adverse change in 22 (18.2%) of the participants. The participants' positive and negative changes in online training were 29 (61.6%) and 18 (38.4%), respectively.

As for the nondominant hand 9-HPT score, F2F education caused a positive change in 95 (78.5%) and an adverse change in 26 (21.5%) of the participants. The positive change was in 27 (57.4%) of the participants for distance education, and the negative change was in 20 (42.6%) of the participants (Table 2).

Based on both dominant and nondominant hand 9-HPT scores, it was observed that there was a statistically significant difference between the change in scores according to the fall/spring term measurements in face-to-face education (*p* < 0.001). Similarly, the dominant and nondominant hand 9-HPT scores showed a statistically significant difference among the change in scores in fall/spring terms in distance education (*p* < 0.001) (Table 2).

The score changes among the 9-HPTs were evaluated for both the dominant and nondominant hand to compare F2F and distance education. The students in the F2F-educated group showed better psychomotor skill development than those in the online-educated group in 9-HPT, and the results showed a statistical significance for both dominant and nondominant hands (*p* < 0.001) (Table 3).

## 4. Discussion

As in all medical fields, anatomy instructors tried to adapt the curriculum to suit the conditions of the COVID-19 pandemic [22, 23]. In this period, the main challenge has been to shift the teaching environment to online mode. Such a transition has become inevitable for medical education programs worldwide, and necessary measures have to be quick [24].

**Figure 2:**
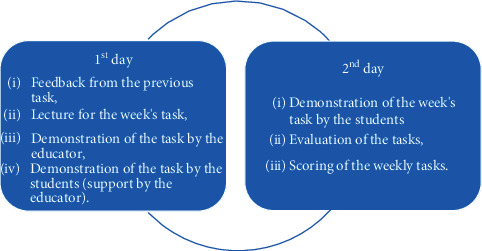
Program weekly training skill schedule for the dental anatomy course during the F2F education period.

At Süleyman Demirel University, the dental anatomy course was being conducted face-to-face (F2F) education (before the COVID-19 pandemic). This course consisted of theoretical and practical components and prioritized psychomotor skill development. The theoretical part was generally based on lectures accompanied by two-dimensional visual materials. The training part was designed to improve the students' psychomotor skills. The course protocols in the F2F education period consisted of theoretical and practical parts and were administered twice a week in the 2019-2020 academic year (Figure 2). The assessment-evaluation protocol was carried out with a focus on practical training. Weekly practical tasks and practical-theoretical exams, which were based on add-on or cutback techniques completed in wax, gypsum, and soap blocks due to relatively low cost, easy handling, and reproducibility, determined the students' academic success [1, 2, 5].

**Figure 3:**
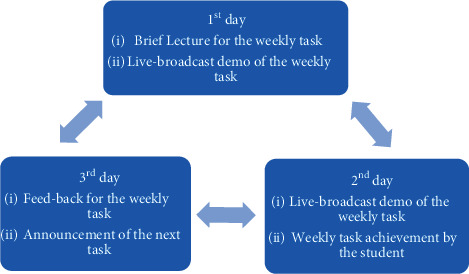
Program weekly training skill schedule for dental anatomy course during the distance education period.

Due to the COVID-19 pandemic in the 2020-2021 academic year, the course was decided to sustain in an online learning environment, and the educational strategy was changed to a flipped-classroom approach. As it is known, one of the outputs of the applied dental anatomy course is the development of psychomotor skills. For this reason, enabling students to make progress in psychomotor skills by using unfamiliar materials has become one of the most critical problems of the distance education process. The distance education protocol implemented during the COVID-19 pandemic created a challenging training process, especially for the preclinical courses in the dentistry curriculum. In addition, the lack of direct communication with the instructor and the lack of in-class interaction brought motivation problems for both the instructors and the students. Distance education was conducted, and a modified online training program was developed with a flipped-classroom approach to overcome these problems during the COVID-19 pandemic. The learning source, including theoretical notes, video records of both anatomical structures, and carving or modeling methods on the checklists, was shared with the students. After they had been given time to increase their readiness and experiment, the instructor performed the live broadcast of the weekly task demo. Furthermore, students were asked to try and demo the weekly tasks too. After they felt self-efficacy, they sent their records of weekly tasks (in time of achievement, they had a chance to communicate with the instructor and take just in time information). Moreover, at the end of the week, they got feedback and scores of weeks (Figure 3).

A studio environment with a special camera was created for the practical course to be demonstrated and taught. All demonstrations throughout the curriculum flow were carried out through this system and broadcast live on the internet. The demonstrations were recorded and made available to the students in a format they could access whenever they wanted. Unlike the F2F training conducted twice a week, distance training was available three days a week during the COVID-19 pandemic (Figure 3).

The demonstrations in the form of live broadcasts formed the basis of this practical course. A WhatsApp group was established with the students of the course. The students accomplished the weekly tasks at home and sent photos and video images to the instructor. The students took the tasks' photos with a caliper for measurement and ratio relationships. They also took video footage for detailed examination by the instructor. Immediate feedback and just-in-time information by the instructor were provided for each assignment. The assignments' feedback was provided visually by marking the images followed by a written report. Practical exams related to this course were also conducted and evaluated as described. The students were free to send their instant task studies to be evaluated by the instructor.

In Turkey, the Council of Higher Education (CoHE) determines the admission criteria for Dentistry Faculty. There are no psychomotor skill tests or interviews among the entry requirements to Dentistry Faculty. The success of the high school graduate students in the university entrance exam and the preference order is sufficient for admission to the faculty. In addition, there are no courses in the primary, secondary, and high school curricula that aim to develop students' psychomotor manual skills. Therefore, it was assumed that the student groups compared in the study were homogeneous in terms of psychomotor skills.

The assessment of students' psychomotor skills during admission to the Faculty of Dentistry or at various stages of dental education has been a source of interest for educators and researchers. Among these tests, no method has been particularly recommended to evaluate psychomotor skills or proficiency for dentistry students [2]. The 9-HPT was preferred due to its ease of application and simplicity of measurement-evaluation procedures [11, 12, 25].

Haptic devices have changed educational practice and environments, especially preclinical education practices [13]. However, before the COVID-19 pandemic, no studies had shown that dental anatomy courses could be taught via online training methods. This introductory dentistry course was mainly given in this country within F2F training protocols as in the rest of the world. Therefore, the authors of this study sought to develop a distance learning protocol to contribute to students' psychomotor skill development and provide course outcomes [26]. The main objective of this study was to assess whether the dental anatomy course given with online training protocols improved psychomotor skills during the COVID-19 pandemic. The data analyzed in this study showed that the online training method applied during the COVID-19 pandemic improved the psychomotor skills of the students. The present study showed satisfactory results for a period when F2F education could not be realized due to mandatory reasons. Compared with the data of the previous (F2F education) academic year's 9-HPT scores, the results of this study also showed that the online training method did not lead to sufficient development in terms of psychomotor skills.

Due to the COVID-19 pandemic, emergency conditions were urged to change the existing education program into an online training environment. In this period, online training opportunities have modified the current F2F course program not to deprive students of education. The online training course program defined in this study was implemented for the 2020-21 academic year.

Face-to-face education has been accepted as a gold standard, especially for applied education. However, the COVID-19 pandemic has introduced some educational practices into our daily lives that have never been employed before. In this study, the existing F2F education protocols were modified in a situation where F2F education was not possible for compulsory reasons. The training skill education of the dental anatomy course was carried out using online training opportunities. This study aimed not to suggest an alternative for F2F education. However, the study and its results can create a starting point or discussion environment when F2F education cannot be performed for various compulsory reasons.

Due to the COVID-19 pandemic, the participation of the students was limited. Voluntary and limited participation, the inability to include all elements of online training in the program, and the short implementation period were among the study's limitations. However, presenting such an educational program for the first time in this field was not without benefits.

## 5. Conclusion

Although face-to-face education is accepted as the gold standard in psychomotor skill training, in case of its impossibility for various reasons, practical training protocols modified for online training may contribute to the motor development of students.

## Figures and Tables

**Table 1:** Descriptive statistics of 9-HPT scores according to the F2F vs. distance education and the dominant hand vs. nondominant hand scores.

	Education protocol	Measurement stage	*n*	Minimum (sec)	Maximum (sec)	Mean (sec)	Std. deviation (sec)
Dominant hand	F2F	1^st^	121	14.80	27.90	20.54	2.48
		2^nd^	121	13.55	27.02	18.31	1.84
	Distance education	1^st^	47	14.80	25.90	20.54	2.50
		2^nd^	47	16.41	24.15	20.09	1.62
Nondominant hand	F2F	1^st^	121	18.10	33.40	22.78	2.65
		2^nd^	121	14.93	33.58	20.52	2.52
	Distance education	1^st^	47	18.20	33.40	22.85	2.91
		2^nd^	47	17.45	26.48	22.01	2.23

**Table 2:** Comparison of the differences between measurements in dominant and nondominant hands in F2F and online training protocols.

	Education protocol	9-HPT measurement (seconds)		*p*	Positive change *n* (%)	Negative change *n* (%)	Positive change mean ± SD	Negative change mean ± SD	*p*
Dominant hand	F2F	1st measuring	20.55 ± 2.48	<0.001^∗^	99 (81.8%)	22 (18.2%)	3.01 ± 1.95	−1.26 ± 1.09	<0.001^∗^
		2nd measuring	18.31 ± 1.84						
	DE	1st measuring	20.54 ± 2.47	<0.001^∗^	29 (61.7%)	18 (38.4%)	1.92 ± 1.99	−1.82 ± 1.46	<0.001^∗^
		2nd measuring	20.10 ± 1.62						
Nondominant hand	F2F	1st measuring	22.78 ± 2.66	<0.001^∗^	95 (78.5%)	26 (21.5%)	3.33 ± 1.9	−1.67 ± 1.77	<0.001^∗^
		2nd measuring	20.52 ± 2.52						
	DE	1st measuring	22.86 ± 2.91	<0.001^∗^	27 (57.4%)	20 (42.6%)	3.33 ± 3.10	−2.51 ± 2.27	<0.001^∗^
		2nd measuring	22.01 ± 2.24						

^∗^Mann–Whitney *U* test.

**Table 3:** F2F vs. online training education comparison due to the 9-HPT score changes.

	Change in the dominant hand	*p*	Change in the nondominant hand	*p*
Face-to-face education	2.23 ± 2.46	<0.001	2.25 ± 2.79	<0.001^∗^
Online training	0.47 ± 2.57		0.84 ± 4.01	

^∗^ *t*-test.

## Data Availability

The [data type] data used to support the findings of this study are included within the article.
